# Exploring the Concept, Antecedents, and Consequences of Environmental Psychological Ownership

**DOI:** 10.3390/ijerph191912621

**Published:** 2022-10-02

**Authors:** Shengxiang She, Shicheng Li, Jiaqi Xu, Bo Yang

**Affiliations:** 1 School of Business Administration, Guizhou University of Finance and Economics, Guiyang 550025, China; 2 Green Development Strategy High-End Think Tank of Guizhou in China, Guiyang 550025, China; 3 Center for Behavior and Decision, Shaanxi University of Technology, Hanzhong 723001, China; 4 Economy School, Zhengzhou University of Aeronautics, Zhengzhou 450015, China

**Keywords:** pro-environmental behavior, environmental psychological ownership, pro-environmental investment, environmental self-efficacy, environmental knowledge

## Abstract

This paper extends the concept of psychological ownership to the general natural environment, clarifies the concept of environmental psychological ownership, and analyzes the formation mechanism of environmental psychological ownership from three dimensions. According to the results of structural equation model based on data obtained from the questionnaire survey, pro-environment investment, environmental self-efficacy, and environmental knowledge are all positively associated with the individual’s environmental psychological ownership, among which the correlation between environmental knowledge and environmental psychological ownership is the strongest. In addition, the environmental psychological ownership positively predicts the individual’s pro-environmental intentions. The conclusion of the study can guide how to strengthen the environmental psychological ownership, thus providing a new perspective for pro-environmental behavior intervention.

## 1. Introduction

The increasingly serious environmental problems are greatly threatening the sustainable development of human society. Therefore, taking action to protect the natural environment has been recognized as one of the most urgent tasks of mankind by all countries in the world. Although almost everyone can realize the importance of environmental protection, there is a widespread gap in attitude and behavior due to various reasons [1]. Especially when individuals are faced with environmental targets involving common interests, due to unclear ownership and decentralized responsibilities, it is difficult to translate positive environmental attitudes into pro-environmental behaviors, resulting in a “tragedy of the commons” [2,3].

Recent studies have advocated the key role of psychological ownership in tackling the barriers for pro-environmental behaviors [1]. Environmental psychological ownership has the potential to strengthen the bond between individuals and the environment, so as to bridge the gap between attitudes and behaviors, enhance the sense of efficacy and responsibility, increase the perceived benefits of pro-environmental behavior, and make the outcomes of environmental actions more relevant [1].

The study of psychological ownership in the context of environment has just started, and the limited studies have mostly focused on the positive role of psychological ownership of specific environmental targets [3,4,5,6,7]. Little is known about the concept of psychological ownership of the general environment, especially the formation mechanism and its consequences. Thus, it is important to explore potential mechanisms that induce psychological ownership for abstract and ubiquitous systems such as the environment, as well as the role of environmental psychological ownership in promoting pro-environmental behaviors. All behaviors that consciously seek to minimize the negative impact on the natural environment are considered to be environmentally beneficial. Almost all people’s behaviors can be related to the natural environment; thus, how to promote general environment-friendly intentions and extensive pro-environmental behaviors is the focus of scholars, which is reflected in most empirical literature in the environmental field, which takes general pro-environment behavior (typically including a dozen of specified environment-friendly actions that differ in terms of their main intent) as the dependent variable, trying to reveal its influence mechanisms. Environment-friendly behaviors entail a variety of different actions, including public observable environment-friendly behaviors, such as participating in volunteer activities, less public observable behaviors, such as signing petitions, making donations for pro-environmental causes, and daily behaviors in the private sphere that also have an impact on the environment, such as waste separation, and the choice of green travel modes [8]. This shows that environment-friendly behavior has many manifestations including a large number of small acts, rather than a single behavior. This makes solving environmental problems a formidable challenge. However, the psychological ownership of a certain environmental target can only have a positive impact on the target, such as a park or a tree, and only general environmental psychological ownership is able to promote extensive pro-environmental behaviors, which is the focus of this study. Therefore, this study is different from the past which only focused on specific psychological ownership. We resolve to explore the concept of psychological ownership and its influence mechanism from the perspective of the general natural environment.

The aim of this study is to clarify the concept of general environmental psychological ownership and analyze its relationships with three antecedents, namely, pro-environment investment, environmental self-efficacy, and environmental knowledge, and then conduct an empirical test through questionnaires and structural equation models. Furthermore, we explore the potential impact of environmental psychological ownership on pro-environmental intentions. Lastly, on the basis of the findings of the study, the policy implications are discussed.

### 1.1. The Concept of Psychological Ownership

Psychological ownership is a subjective sense that a target belongs to oneself and is embodied in the expression “this is mine!” [9]. Such feelings of ownership can easily exist in the absence of legal rights. Theoretically, feelings of ownership can be experienced for anything, including tangible objects such as cups or pens [10], something rather intangible like a brand [11], an investment [12], or a job [13], something as abstract as an idea [14]. Psychological ownership can also emerge for common goods such as the environment or aspects of it [15]. In fact, Sussenbach and Bernadette [1] found that people report feelings of ownership for the environment and that there are substantial variations in the degree to which people experience these feelings.

The term environment is widely used in academic expression, as well as in daily life. Yet, in discourses of sustainability, the term is meant to refer to a system in which the whole of humanity is embedded. By environment, we mean “the natural world as a whole […]” and “the surroundings or conditions in which a person, animal, or plant lives or operates” [16]. The natural environment includes all living things and natural elements such as air, water, soil, or climate in contrast to the built environment [17]. It comprises all elements “which, in their complex interrelationships, form the framework, setting, and living conditions for mankind, by their very existence or by virtue of their impact” [18]. It is the natural environment in its entirety that we refer to as “the environment”.

This study proposes that individuals will not only have psychological ownership of specific environmental objects such as lakes and parks, but also have overall psychological ownership of the general natural environment, which has a broader and far-reaching impact on individuals’ pro-environmental behavior. Human beings originate from nature, live in the natural environment, and finally return to nature. Therefore, individual psychology should contain a certain synergy with the environment, which is reflected in the individual’s cognitive emotional structure of awareness, ideas, and beliefs about the environment. Especially for Chinese people, the core values of Chinese traditional culture, such as “nature” and “harmony”, are the historical genes passed down from generation to generation in Chinese culture, which are embodied in the spiritual domain of Chinese people as the ultimate ideal [19]. Qian Mu [20], a famous modern Chinese historian, thinker, and educator, believes that the concept of “the unity of heaven and man” is the most contributory proposition of ancient Chinese culture. Obviously, Chinese people have always pursued the harmony and unity between man and nature.

The study of pro-environmental behavior pays attention to both specific environmental behavior and general pro-environmental behavior [8,21]. Therefore, theoretically, there is a corresponding concept of general environmental psychological ownership. In fact, Felix and Almaguer [15] mentioned the psychological ownership of the earth, although the concept of environmental psychological ownership was not clear, nor were its antecedents studied.

In this study, environmental psychological ownership in a general sense is defined as individuals’ subjective feelings that the surrounding natural environment belongs to “themselves” on the whole; thus, they feel that, to some extent, they are the “master” or “manager” of the environment. This study reveals the formation mechanism of environmental psychological ownership and its impact on general pro-environmental behavior through empirical research.

### 1.2. Antecedents of Environmental Psychological Ownership

According to Pierce et al. [19], three essential routes may lead to the development of psychological ownership, i.e., perception of control, personal investment, and intimate knowledge of an object. Translated to the context of the environment, this means that any perceived increase in control over the environment, any perceived increase in knowledge about the environment, and any personal investment that people perceive to make into the environment may help to instigate some sense of ownership for it.

#### 1.2.1. Pro-Environmental Investment

Early studies pointed out the potential impact of personal investment on the psychological ownership of specific objects. For example, Locke [22] believed that people associate their labor with the resulting creation, as well as have a sense of ownership of the things they create, produce, and shape. Kirkpatrick [23] clearly proposed that individuals can achieve the integration of self-concept and things through self-investment. Recent empirical studies on consumer psychological ownership found that providing text and picture content for product design and other behaviors that require time and energy lead to customers’ psychological ownership of customized products [24]. Furthermore, by creating playlists and self-made music collections, consumers have formed the psychological ownership of streaming media music [25].

In the context of environmental protection, any pro-environmental activities implemented by individuals in the past required a certain degree of personal investment. Specifically, personal investment includes the time, money, thoughts, skills, and physical, psychological, and intellectual energy paid by individuals [26]. For example, many people struggled for environmental protection in various ways for a long time. These pro-environmental investments not only affect the sense of ownership of specific environmental targets, but may also be extended to the general natural environment. Therefore, the more individuals invested in environmental protection in the past, the stronger their psychological ownership is of the natural environment. The following assumption is proposed:

**H1.**  *Individual pro-environment investment is positively associated with environmental psychological ownership.*

#### 1.2.2. Environmental Self-Efficacy

Environmental self-efficacy is the belief held by individuals about their ability and degree to curb environmental degradation [27], i.e., to what extent individuals believe that they can change the situation of environmental damage and pollution. Self-efficacy can induce psychological ownership by changing the perception of the state and attributes of the target [9]. Even imagined or potential control over an object leads to enhanced perceptions of ownership [10]. Given that perception of control lies at the heart of perceived efficacy [28], individuals with a stronger sense of environmental self-efficacy believe that their pro-environmental behavior can improve the environment, and they have a stronger sense of control over the environment, such that they can feel a stronger sense of ownership for the environment. Pierce et al. [9] pointed out that self-efficacy is one of the motives for individuals to obtain psychological ownership of something. Therefore, individuals with stronger environmental self-efficacy have more motivation to regard the natural environment as “theirs”. The following assumption is proposed:

**H2.**  *Individual environmental self-efficacy is positively associated with environmental psychological ownership.*

#### 1.2.3. Environmental Knowledge

Environmental knowledge is defined as the degree to which people express concern and understanding about the natural environment [29]. According to Pierce et al. [9], another key route of psychological ownership is to integrate the target into self-concept through intimate knowledgeable about the target. Knowing and being familiar with things is one of the ways for individuals to integrate things into themselves [30]. Beggan and Brown [31] also emphasized the role of the relationship between individuals and objects in the sense of ownership. In other words, individuals will feel that an object is “theirs” because they are familiar with it. The increase in the amount of information about an object will deepen the individual’s understanding of the object, the relationship between the individual and the object will also deepen, and the feeling of psychological ownership will be stronger. For example, Slater [32] found that consumers’ psychological ownership of the Coca Cola brand largely stems from their deep understanding of the brand. The knowledge and familiarity with music platforms and social platforms stimulate consumers’ psychological ownership for the platform [25]. Accordingly, with the increase in environmental knowledge, people will have a deeper understanding of environmental problems, so as to improve the relevance between themselves and the natural environment, and then strengthen the sense of environmental psychological ownership.

Sussenbach and Bernadette [1] suspected that perceived environmental knowledge might be the most powerful antecedent in producing psychological ownership for the environment. One can feel knowledgeable about the environment via multiple avenues. This perception of environmental knowledge could enhance sense of ownership for the environment and, finally, result in more pro-environmental behavior. Currently, environmental knowledge is widely available, and almost all environmental knowledge can be collected and transmitted to the general public. Therefore, knowledge about the environment could be most promising in instigating psychological ownership for the environment and, thus, pro-environmental behavior. Knowledge is a vague concept, involving a variety of aspects of the environment, as well as how to engage in pro-environmental activities [33]. Moreover, it can be actual knowledge or perceived knowledge [34].

**H3.**  *Individual perceived knowledge about the environment is positively associated with environmental psychological ownership.*

### 1.3. The Relationship between Environmental Psychological Ownership and Pro-Environmental Behavior

When an individual has psychological ownership of something, she or he will regard the psychological possession as a part of self-concept [9], thus stimulating more active personal sacrifice and risk-taking, as well as greater responsibility taking and protection behaviors [35]. In specific situations, existing studies have shown that psychological ownership can promote personal pro-environmental behavior. For example, Peck et al. [3] found that kayak tourists picked up more lake garbage after their psychological ownership of the frequently visited lakes was improved, while tourists with psychological ownership of the park donated more money to protect the park. Another study found that individuals with stronger psychological ownership of the earth showed stronger intention to recycle garbage and purchase green products [4]. Therefore, this study suggests that individuals with stronger environmental psychological ownership are willing to take more extensive pro-environmental behaviors in order to protect the natural environment.

**H4.**  *Individual environmental psychological ownership is positively associated with pro-environmental intentions.*

On the basis of above hypotheses, this study further suggests that environmental psychological ownership plays a mediating role between the three antecedents and pro-environmental behavior.

**H5.**  *Environmental psychological ownership mediates the relationship between the three antecedents and pro-environmental intentions.*

**Figure 1:**
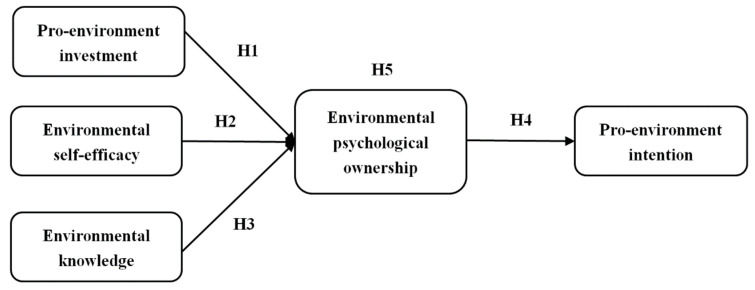
Research model.

Finally, we get the conceptual model of this study, as shown in Figure 1.

## 2. Materials and Methods

### 2.1. Sample

All people have more or less psychological ownership of their natural environment. The object of this study is an ordinary person in China. Our main purpose was to verify the concept of environmental psychological ownership and its mechanisms; accordingly, we did not intend to conduct a sample survey representing all Chinese individuals. In this study, Credamo, similar to Mturk, was used to design a questionnaire and obtain data. Using this professional online questionnaire design and survey platform, we designed an electronic questionnaire and purchased 550 Chinese samples through the sample database of Credamo. We did not specify any sample features; hence, the samples were randomly selected by the platform. According to the attention test question inserted into the questionnaire “No matter what the weather is like outside, you should keep a good attitude. Please choose ‘3’ for this statement”, 54 invalid questionnaires were deleted, and 496 valid questionnaires were finally obtained. Among them, women accounted for a slightly higher proportion, 54.2%; 21 to 30 years old accounted for a large proportion (55.8%, 277 people). Most respondents (71.2%, 353 people) had a bachelor’s degree, working in private enterprises (37.7%, 187 people) or state-owned enterprises (25%, 124 people). Detailed sample characteristics are shown in Table 1.

### 2.2. Measure

Pro-environment investment was measured with the question “Did you often make efforts (such as spending time, energy or money, etc.) to implement the following pro-environmental behaviors? (1 = strongly disagree, 7 = strongly agree)”. The five items were adapted from Kaiser and Wilson [36]; an example is “reuse plastic bags”.

Environmental self-efficacy was measured with the question “To what extent do you agree with the following statements? (1 = strongly disagree, 7 = strongly agree)”. The four items were adapted from Earley et al. [37] and Tabernero and Hernández [38]; an example is “I think that I have the ability to participate in actions to protect the natural environment”.

Environmental psychology ownership was measured with the question “For the natural environmental you are live in, to what extent do you agree with the following statements? (1 = strongly disagree, 7 = strongly agree)”. The four items were adapted from Fuchs et al. [39]; an example is “I feel this is my natural environment”.

Pro-environment intention was measured with the question “In order to protect the natural environmental you live in, to what extent are you willing to implement the following environmental behaviors? (1 = strongly disagree, 7 = strongly agree)”. The 11 items were adapted from Sun [40]; an example is “buy products whose packaging is marked as reusable, recyclable, or renewable”. See Appendix A for the complete content of the scales.

Notably, the measurement of environmental knowledge includes two measures, where subjective environmental knowledge was measured using two items adapted from Stuart [41], such as “To what extent do you believe that you are informed about various natural environment issues? (1 = strongly disagree, 7 = strongly agree)” and “To what extent do you believe that you are knowledge about natural environment? (1 = strongly disagree, 7 = strongly agree)”. The objective environmental knowledge was measured using nine questions adapted from Hong et al. [42] and Carmi et al. [43] (see Appendix B). If the answer was correct, one point was counted; if the answer was wrong or “have no idea” was selected, 0 points were counted. The scores of nine questions were summed as an indicator of environmental knowledge. The range of this indicator was 0–9, where a larger value indicates more objective environmental knowledge.

## 3. Results

### 3.1. Reliability and Validity Test

Confirmatory factor analysis (CFA) was implemented to evaluate the adequacy of the constructs for the five adopted measurements. As shown in Table 2, all factor loading was significant (*p* < 0.001), ranging from 0.63 to 0.86. Composite reliability (CR) for the constructs ranged from 0.86 to 0.91, and Cronbach’s alphas were above the benchmark of 0.7, suggesting reliable measures. The average variance extracted (AVE) of all constructs exceeded the recommended value of 0.50, ranging from 0.55 to 0.67. The discriminant validity of the constructs was assured as the AVE of each construct exceeded its squared correlation to any other construct.

### 3.2. Common Method Variance

There is a possibility of common method bias (CMV) in self-reported questionnaires. A CMV between data suggests a false relationship between constructs. Credamo, used in this study to collect data, is a professional platform with a high-quality sample base. Credamo provides multiple data quality assurance through screening questions and review strategies, and gives five mandatory reading tips before the questionnaire starts to ensure that the respondents carefully answer the questions. Furthermore, Harman’s single-factor method was used to test the CMV. Specifically, using exploratory factor analysis, if the first factor variance interpretation of the first factor exceeds 50%, it will have a higher CMV. The initial eigenvalue of the first factor in this study was 38.09%; hence, this study was not threatened by CMV.

### 3.3. Structural Model

The correlation analysis showed significant positive correlations between psychological variables, suggesting an internal relationship between these variables. As for demographic variables, only age had a slight positive correlation (0.11–0.24, see Table 3) with the five variables. According to age characteristic of our sample, we divided them into the lower age group (30 years and younger) and the higher age group (31 years and older) and conducted a comparison analysis. The sample *t*-test shows that, with the increase in age, pro-environmental investment (MHigher = 6.01, SD = 0.69; MLower = 5.66, SD = 0.79, *t* = 5.07, *p* = 0.00), environmental knowledge (MHigher = 6.39, SD = 0.98; MLower = 6.11, SD = 1.07, *t* = 2.90, *p* = 0.003), environmental psychological ownership (MHigher = 5.69, SD = 0.74; MLower = 5.37, SD = 0.90, *t* = 4.27, *p* = 0.00), and pro-environmental intentions (MHigher = 5.91, SD = 0.70; MLower = 5.57, SD = 0.79, *t* = 4.95, *p* = 0.00) were significantly increased. However, the difference in environmental self-efficacy between the lower and higher age groups was not significant (*t* = 1.87, *p* = 0.062).

We adopted PLS-SEM to estimate the structural model. PLS-SEM is appropriate as it does not assume normal distributions of data [44] and SmartPLS 3.0 can provide consistent results when all constructs are reflective. If used properly, PLS-SEM can provide more robust estimations of structural models than covariance-based SEM. The significance of the model estimates was based on a bootstrapping procedure with 5000 samples. The model fit was ideal (SRMR = 0.06 < 0.08, RMS-theta = 0.11 < 0.12, d_G = 0.42 < 0.95).

**Figure 2:**
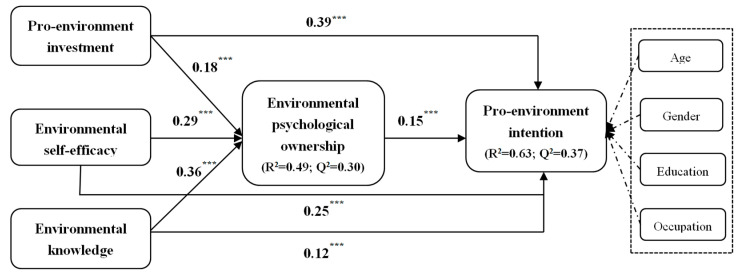
The estimates of SEM. Note: *** *p* < 0.001.

As shown in Figure 2, pro-environmental investment (β = 0.39, *t* = 8.13, *p* < 0.001), environmental self-efficacy (β = 0.25, *t* = 4.82, *p* < 0.001), and environmental knowledge (β = 0.12, *t* = 3.01, *p* = 0.002) positively predicted pro-environmental intentions. Furthermore, pro-environmental investment (β = 0.18, *t* = 3.59, *p* < 0.001), environmental self-efficacy (β = 0.29, *t* = 5.30, *p* < 0.001), and environmental knowledge (β = 0.36, *t* = 8.05, *p* < 0.001) were positively associated with environmental psychology ownership, supporting H1, H2, and H3, respectively. Furthermore, environmental psychology ownership was positively associated with pro-environmental intentions, supporting H4.

### 3.4. Mediation Analysis

To test whether the relationship between antecedents and pro-environmental intention was mediated by environmental psychology ownership, we used the bootstrap confidence interval test method. Table 4 shows the results of the mediating effects of environmental psychology ownership in this study. Results shows that environmental psychological ownership played a partial mediating role between pro-environment investment (β = 0.027, *t* = 2.34, *p* = 0.021), environmental self-efficacy (β = 0.014, *t* = 2.45, *p* = 0.014), and environmental knowledge (β = 0.054, *t* = 3.08, *p* = 0.003) and environmental intention, supporting H5.

## 4. Discussion

This study explored the concept of environmental psychological ownership, and investigated the motivation factors of pro-environmental behavior from a general perspective. The existing research on psychological ownership mainly occurred in the fields of organizational behavior and consumer behavior. The relevant research in the field of pro-environmental behavior is very limited, and these studies only extended the concept of psychological ownership to specific environmental objects. For example, Preston and Gelman [5] and Peck et al. [3] activated the individual’s psychological ownership of the park or lake by letting them read an essay or name the target. The traditional Chinese culture regards nature as a whole and teaches people that they should keep the world in mind and integrate heaven and man. This concept has long and deeply influenced Chinese people from generation to generation. Therefore, we proposed and verified for the first time that people can also have psychological ownership of the general natural environment, substantially expanding the research scope of psychological ownership. From the perspective of ecology and system theory, the Earth is an ecological giant system in which all elements are closely linked and interacted. This concept has also been the consensus of people all over the world. Therefore, we believe that the findings of this study are universal and can provide inspiration for practitioners engaged in environmental research and practice. Existing relevant studies focus on some specific environmental contexts such as lakes and parks [3,4,5,6,7]. Although people have stronger psychological ownership of specific environmental objects, the positive impact of this narrow sense of psychological ownership is also limited to specific environmental objects, with limited practical significance. However, it is reasonable to believe that general environmental psychological ownership and specific environmental psychological ownership have some positive interaction, because a specific environmental object is part of the overall environment. Therefore, the research findings on these two aspects can support and verify each other.

In addition, this study revealed the effects of the three key factors—pro-environmental investment, environmental self-efficacy, and environmental knowledge—on environmental psychological ownership, which provides a theoretical perspective and evidence for understanding the formation mechanism of general environmental psychological ownership. At present, the research on the antecedents of environmental psychological ownership is weak. Although Sussenbach and Bernadette [1] claimed that environmental knowledge is a potential antecedent contributing to the improvement of environmental psychological ownership, they provided no empirical evidence. Some studies successfully activated the psychological ownership of specific environmental targets by using experimental methods, such as letting participants read a short essay about a natural area (forest, lake, or garden) [5], increasing their understanding of the natural area, or asking participants to name a lake or plan the touring route of a park [3], leading to their self-investment. These studies provide evidence for the systematic study of the antecedents of environmental psychological ownership, consistent with the results of this study. Our research shows that a person’s daily environmental efforts, understanding of environmental knowledge, and confidence in self ability can predict the psychological ownership of the general natural environment. It is worth noting that the correlation analysis and *t* test implied a positive correlation between age and environmental psychological ownership and its antecedents, indicating that environmental self-investment and environmental knowledge will increase with age and, thus, the psychological ownership of the environment will be stronger. Although the impact of age was not the focus of this study, this finding further shows that the individual’s psychological ownership of the general environment requires time and experience.

Consistent with the existing results, this study verified the positive role of the general environmental psychological ownership in pro-environmental behavior intentions. Previous studies theoretically discussed how psychological ownership affects environmental protection behavior. For example, Sussenbach and Bernadette [1] stated that psychological ownership can promote a variety of pro-environmental behaviors, but no empirical research has been carried out. Some recent empirical studies showed that psychological ownership of specific environmental target can indeed promote specific pro-environmental behaviors. For instance, Preston and Gelman [5] found that activating subjects’ psychological ownership of specific natural reserve triggered stronger protection intention than legal ownership. Peck et al. [3] confirmed the positive role of psychological ownership on environmental protection behavior by manipulating individuals’ psychological ownership of lakes and parks. These results provided important guidance for this study. On this basis, this study reveals for the first time the positive relationship between general environmental psychological ownership and a series of environment-friendly behavioral intentions, which proves that general environmental psychological ownership has the ability to strengthen more extensive pro-environment behaviors. Therefore, this study provides new insights for identifying the influencing factors of pro-environmental behaviors.

## 5. Implications and Limitations

According to the results of this study, strengthening the individual’s environmental psychological ownership can promote pro-environmental behavior. Therefore, it is of great practical significance to strengthen the internal motivation of people to take pro-environmental behavior from the perspective of psychological ownership. This study confirms that pro-environmental investment, environmental self-efficacy, and environmental knowledge can positively affect individual environmental psychological ownership, which provides useful enlightenment for government departments and environmental protection organizations to promote public pro-environmental behavior.

Firstly, we should create as many opportunities as possible for the public to participate in various environmental protection practices and encourage them to make pro-environmental investments. For example, stakeholders are invited to discuss environmental protection issues such as rivers, lakes, soil, and garbage disposal, so as to enhance their psychological ownership of the ecological environment. Furthermore, encouraging environmental organizations to launch a large number of low-threshold environmental volunteer activities can guide the public to contribute their energy and time. In the digital age, people’s daily environmental protection behaviors can be converted into points by means of self-quantification, which can highlight the environmental protection efforts that individuals have made in the past, thus helping to strengthen individual environmental psychological ownership. The “Ant Forest” mobile phone application launched by the Alibaba Group provided a good reference. Its users’ daily low-carbon life behavior is digitized into green energy that can be accumulated continuously, and then transformed into actual saplings planted in desert areas. Correspondingly, the users obtain an environmental protection certificate marking their achievements in mobile applications, which can constantly remind users of their efforts toward a green low-carbon planet. Obviously, such digital innovation means can induce more investment in environmental protection and help enhance people’s psychological ownership of the environment.

Secondly, in order to improve the environmental self-efficacy and further strengthen the public’s environmental psychological ownership, environmental organizations can publicize the past success stories of environmental protection to enhance the public’s confidence in solving environmental problems. Government departments can solicit and adopt public opinions in the process of environmental governance, so that the public can perceive that they can influence the solution of environmental problems. In particular, all sectors of society should help environmental vulnerable groups or individuals to remove various subjective and objective rights barriers, so that they can perceive their own strength and stimulate their own internal motivation through positive experience, as well as improve the ecological environment on which we depend through environmental engagement. The convenience of public engagement channels determines the launch and participation frequency of public environmental engagement to a certain extent. Online participation in the information age can effectively break through the space–time bottleneck of public environmental engagement, as well as provide a platform beyond time and space for the broadest public engagement. For example, government decisions on environmental issues should be open and transparent through the Internet, so that the public can find problems and put forward their own demands. The network can also avoid the psychological cowardice and embarrassment that people are prone to when communicating face to face, which makes the ordinary public increase their courage to express themselves in the process of supervision. In addition, online participation can allow public supervision of the whole process.

Lastly, various measures can be taken to promote the public’s understanding of the ecological environment and connect people with the nature. In addition to further standardizing, popularizing, and strengthening environmental education in schools at all levels, governments and social organizations need to mobilize social elites to continuously output knowledge to the general public through certain platforms, such as television and the Internet. In particular, we should give play to the role of environmental Non-Governmental Organizations (NGOs), because NGO members not only have a strong sense of environmental protection, but also have relatively professional environmental knowledge; thus, they can give professional help and support to the environmental protection activities of the general public. In practice, in addition to creating opportunities for people to have more contact with nature, such as holding environmental science publicity, we can spread environmental knowledge by promoting serious games [45]. In September 2019, the United Nations Climate program launched the “playing for the planet” alliance at the climate summit, calling on the world’s game industry to join hands and encourage more players to participate in the action of environmental protection. However, there is still substantial room for the development of environmental serious games in China.

This study inevitably had some limitations. First of all, this study adopted the self-report method when collecting data. Although this is a common method to study attitudes, mental states, and behaviors, we must note the potential social desirability bias when interpreting the results. Secondly, this study used pro-environmental intention instead of pro-environmental behavior. Although this is a common practice in academic research, it must be recognized that there is still a gap between intention and actual behavior. It will be more convincing if we can observe the real pro-environment behavior of the respondents and then link the concept of psychological ownership with it. This study attempted to theoretically establish the relationship between the three antecedents, i.e., pro-environment investment, environmental self-efficacy, and environmental knowledge, and environmental psychological ownership; therefore, the correlation method based on cross-sectional data was used. However, experimental methods need to be used if the causal relationship between variables is to be identified, which is worth further study. Lastly, psychological ownership can be divided into the individual level and collective level. Collective psychological ownership refers to the individual’s perception that the target of ownership is “ours” [46]. This study only discussed the concept and antecedents of environmental psychological ownership at the individual level. The concept and antecedents of collective environmental psychological ownership are worth studying.

## 6. Conclusions

Solving environmental problems is an arduous task, and the challenge of ensuring a sustainable future will ultimately fall on the shoulders of entire public. In order to encourage as wide a range of pro-environmental behaviors as possible, it is necessary to fully release people’s internal motivation to overcome many obstacles. On the basis of the existing literature on psychological ownership, this study proposed the concept of general environmental psychological ownership in the context of Chinese culture. We adopted the theoretical perspective of Pierce et al. [9] on the routes of psychological ownership and analyzed its formation mechanism and its role in promoting pro-environmental intentions. Through empirical research on Chinese people, we verified the positive effect of three antecedents, namely, pro-environmental investment, environmental self-efficiency, and environmental knowledge, on environmental psychological ownership, as well as the mediating mechanism of environmental psychological ownership as causing stronger pro-environmental intentions. The results show that the contribution of environmental knowledge to the formation of environmental psychological ownership is the strongest, followed by environmental self-efficacy. These results provide a perspective for promoting broader pro-environmental behavior through policy or commercial means. Lastly, this paper discussed the implications of these conclusions according to the research findings.

## Figures and Tables

**Table 1:** Demographic characteristics of the sample (N = 496).

Demographic Variable	Category	Frequency	Percentage (%)
Gender	Male	227	45.80
	Female	269	54.20
Age	18–20	28	5.60
	21–30	277	55.80
	31–40	169	34.10
	41–50	17	3.40
	51–60	5	1
Education	Junior high school	4	0.80
	High school	21	4.20
	Junior college	47	9.50
	Bachelor	353	71.20
	Master	66	13.30
	PhD	5	1
Occupation	Student	105	21.20
	Civil servant	12	2.40
	State-owned enterprise	124	25
	Private enterprise	187	37.70
	Government-affiliated institutions	51	10.30
	Foreign enterprise	16	3.20
	Other	1	0.2

**Table 2:** Results of confirmatory factor analysis.

Constructs	Items	Factor Loading	α	AVE	CR
PEI1 (M = 5.80; SD = 0.77)	Item 1	0.78	0.79	0.55	0.86
	Item 2	0.75			
	Item 3	0.71			
	Item 4	0.74			
	Item5	0.72			
ESE (M = 5.90; SD = 0.77)	Item 1	0.80	0.82	0.65	0.88
	Item 2	0.82			
	Item 3	0.80			
	Item4	0.80			
EK (M = 6.22; SD = 1.04)	Item 1	0.86	0.75	0.67	0.86
	Item 2	0.86			
	Object environmental knowledge	0.72			
EPO (M = 5.50; SD = 0.86)	Item 1	0.81	0.82	0.65	0.88
	Item 2	0.80			
	Item 3	0.78			
	Item 4	0.83			
PEI2 (M = 5.70; SD = 0.77)	Item 1	0.63	0.89	0.65	0.91
	Item 2	0.65			
	Item 3	0.66			
	Item 4	0.70			
	Item5	0.70			
	Item6	0.69			
	Item7	0.73			
	Item 8	0.66			
	Item9	0.74			
	Item 10	0.73			
	Item 11	0.73			

Note: Pro-environment investment = PEI1; environmental self-efficacy = ESE; environmental knowledge = EK; environmental psychology ownership = EPO; pro-environment intention = PEI2.

**Table 3:** Construct correlations.

	1	2	3	4	5	6	7
1 PEI1	**0.70**						
2 ESE	0.64 ***	**0.81**					
3 EK	0.50 ***	0.53 ***	**0.82**				
4 EPO	0.56 ***	0.60 ***	0.60 ***	**0.81**			
5 PEI2	0.73 ***	0.67 ***	0.56 ***	0.61 ***	**0.81**		
6 Gender	0.04	0.01	0.06	0.07	−0.02	——	
7 Age	0.24 **	0.11 *	0.13 **	0.17 **	0.23 **	0.01	——

Note: Diagonal elements in bold font are the square root of AVE from their indicators, *** *p* < 0.001, ** *p* < 0.01, * *p* < 0.05. Pro-environment investment = PEI1; environmental self-efficacy = ESE; environmental knowledge = EK; environmental psychology ownership = EPO; pro-environment intention = PEI2.

**Table 4:** Mediation effect.

Effect	Relationship Path	Effect Size	*t*	95% Confidence Interval	
Indirect effect	PEI1 → EPO → PEI2	0.027 *	2.34	0.008	0.051
	ESE → EPO → PEI2	0.045 *	2.45	0.014	0.083
	EK → EPO → PEI2	0.054 **	3.08	0.023	0.091
Direct effect	PEI1 → PEI2	0.39 ***	8.13	0.293	0.481
	ESE → PEI2	0.25 ***	4.82	0.150	0.353
	EK → PEI2	0.12 **	3.01	0.040	0.203

Note: *** *p* < 0.001, ** *p* < 0.01, * *p* < 0.05.

## Data Availability

The data presented in this study are available on request from the corresponding author.

## Appendix A

**Constructs**	**Items**
PEI1	Reusing plastic bags
	Saving water (such as timely closing the tap, water flowers with vegetable washing water, reduce flush of toilet, etc.)
	Using the other side of the written paper
	Encouraging family and friends to save energy and water, as well as participate in recycling
	Use of public transport or bicycles for close travel (within 3 km)
ESE	I think that I have the ability to participate in actions to protect the natural environment
	I am sure that I can do some things to protect the natural environment
	I am confident that I can participate in actions to protect the natural environment
	I believe that I can take actions to protect the natural environment
EK	To what extent do you believe that you are informed about various natural environment issues?
	To what extent do you believe that you are knowledge about natural environment?
	Object environmental knowledge (see Appendix B)
EPO	I feel this is my natural environment
	I feel I own natural environment
	I feel a part of myself is contained in the natural environment
	I feel a strong sense of connection with natural environment
PEI2	Not buying overly packaged products
	Purchasing nontoxic, phosphate-free, and biodegradable soaps and detergents
	Buying products whose packaging is marked as reusable, recyclable, or renewable
	Trying not to use disposable items (plastic bags, disposable tableware, paper towels, paper cups, etc.)
	Bringing own shopping bags or avoiding the use of plastic or paper bags from the store when shopping
	Sorting and recycling waste resources (recycling wastepaper, aluminum cans, glass bottles, plastic bottles, used batteries, etc.)
	Participating in citizen meetings related to environmental issues (party and government meetings, citizen hearings, etc.)
	Letters or visits to the National People’s Congress or government officials to express opinions on environmental issues
	Prosecution of persons or units that destroy the environment to government departments
	Advising others not to violate environmental regulations or informing them that their actions have violated environmental regulations
	Publicly expressing support for environmental protection (forwarding environmental protection information, participating in environmental protection publicity, voting, giving speeches, accepting interviews, etc.)

## Appendix B

Please judge whether the following statements about the environment are true? (true = 1; false or have no idea = 0)

Increased carbon dioxide content in the atmosphere can lead to a warming climate

Species are interdependent, and the disappearance of one species will have a chain reaction

Single species of trees are more susceptible to pests and diseases

In the air quality report, level 3 air quality is better than level 1 air quality

In the water pollution report, water quality of V (5) is better than water quality of I (1)

It takes about 200 years for aluminum beverage cans to decompose in nature

Global warming is caused by the hole in the ozone layer

Rising carbon dioxide levels in the air could exacerbate the hole in the ozone layer

Electric vehicles will greatly reduce our reliance on gasoline, coal, and natural gas
